# Hyperparameter-controlled regularized reconstruction method based on object structure and acquisition conditions in SPECT

**DOI:** 10.1186/s40658-025-00788-7

**Published:** 2025-07-29

**Authors:** Tomoya Minagawa, Kensuke Hori, Takeyuki Hashimoto

**Affiliations:** 1 Department of Radiology, Toho University Ohashi Medical Center, 2-22-36 Ohashi, Meguro-ku, Tokyo 153-8515 Japan; 2 Department of Medical Radiological Technology, Graduate School of Health Sciences, Kyorin University, 5-4-1 Shimorenjaku, Mitaka-shi, Tokyo 181-8612 Japan; 3 Department of Radiological Technology, Faculty of Health Science, Juntendo University, 3-2-12 Hongo, Bunkyo-ku, Tokyo 113-0033 Japan

**Keywords:** SPECT, Regularized reconstruction method, Automatic controlled hyperparameters

## Abstract

**Background:**

In clinical nuclear medicine, reconstruction methods incorporating regularization terms have been widely investigated. However, searching for optimal hyperparameters for the entire examination is time-consuming and arduous because the optimal hyperparameters need to be determined experimentally and vary depending on factors, including the acquisition condition, reconstruction condition, and so on. In this study, we propose a row-action type automatic regularized expectation maximization method (RAREM). This method considers the acquisition conditions and object structure for determining the hyperparameters and does not require the user to set the hyperparameters experimentally. This study was conducted using numerical simulations and a real SPECT system

**Methods:**

Total variation-expectation maximization (TV-EM) and modified-block sequential regularized EM (BSREM) were compared with RAREM, with the optimal hyperparameters of the two conventional reconstruction methods determined in advance from normalized root mean square error (NRMSE) results. This simulation examination utilized three types of phantoms with the number of counts and projections being examined in six ways each, resulting in a total of 108 conditions. The NRMSE and structural similarity index measure (SSIM) were used to evaluate of the simulation examination, and the Mann–Whitney U test was used for statistical analysis. In the real examination, two types of phantoms were used, and the number of projections was examined in three ways, for a total of six conditions. Contrast recovery coefficient (CRC) and specific binding ratio (SBR) were used to evaluate the real examination

**Results:**

The NRMSE, CRC, and SBR of RAREM were equivalent to those of the conventional methods, and the SSIM of RAREM was equivalent to or better than that of the conventional methods, with significant differences in some cases. The results indicated that RAREM worked well with the evaluated object structure and considered the acquisition conditions

**Conclusion:**

In this study, an automatically controlled regularization reconstruction method was proposed. The proposed method does not require the user to set hyperparameters experimentally and can avoid the investigation of optimal hyperparameters; it is an alternative to conventional regularized methods in clinical

## Background

SPECT can be used to obtain functional images, which are important in clinical practice. However, obtaining high-quality images is time-consuming, and SPECT images exhibit a higher level of noise than the other modalities such as CT, MRI, and PET. In particular, compared with PET, which does not use a collimator, SPECT is limited by its low sensitivity and is significantly affected by noise. Furthermore, when the administered dose is low, or a nuclide with a long half-life is used, ensuring an adequate total count is challenging. Hence, the selection of appropriate acquisition conditions and reconstruction methods is important.

Reconstruction methods for overcoming high-level noise have been extensively investigated. Row-action type reconstruction methods introduced relaxation parameter to reduce noise [1, 2]. Particularly in DRAMA, the relaxation parameter is automatically controlled for each sub-iteration, making the noise propagation to the reconstructed image constant [2]. This process improves the signal-to-noise ratio compared to conventional iterative reconstruction methods, such as ML-EM and OS-EM. Nevertheless, image quality is degraded when data have a high noise level.

Another approach to suppress noise is the incorporation of a regularization term as a prior information of images into ML-EM, called the MAP-EM algorithm, which is based on Bayes’ theorem [3]. TV-EM is a typical form of the MAP-EM algorithm that uses a TV regularization term [4]. It can reduce noise and is often used for sparse-view reconstruction [5–8]. In clinical applications, BSREM, which is also a MAP-EM algorithm, used for Q.Clear (GE Healthcare, Milwaukee, WI, USA) is the most commonly used regularized reconstruction method for PET [9–11]. It was reported that the BSREM improved the quality of reconstructed images and lesion detectability [12, 13]. However, several hyperparameters must be determined experimentally. In the modified-BSREM proposed by Ahn et al., the variable parameters are the number of subsets, the coefficient of the regularization term, the initial value of the relaxation parameter, and its rate of decrease at each iteration [10]. Because the optimal parameters vary depending on various factors, such as the acquisition conditions and objective lesion, etc., searching for all optimal parameters requires considerable time and effort [12–15]. Recently, SPECT systems that can use BSREM (or modified-BSREM) have become available [16]. Therefore, in SPECT, the search for optimal parameters for each purpose of examination is expected to increase.

In this study, we propose the automatic reconstruction algorithm, which called the RAREM, to omit the investigation of optimal hyperparameters. This method has two main features. The first is an automatic hyperparameter control system that considers object structure and acquisition conditions. Using spatial filter, the object structure was evaluated based on the edges of the update images. The second is the observance of the non-negativity constraint by considering the value that the regularization term can assume. This study aimed to evaluate whether the performance of RAREM is equivalent to or better than that of conventional regularized reconstruction methods using optimal hyperparameters. The study was conducted using numerical simulations and a real SPECT system.

## Methods

TV-EM and modified-BSREM were compared with RAREM. In this study, scatter, attenuation, and collimator aperture corrections were not used to compare the differences between image reconstruction methods.

### Conventional reconstruction method

In SPECT, the projection data follows the Poisson distribution $P\left( {\varvec{y}}|{\varvec{x}}\right)$.

1 $$\begin{aligned} P({\varvec{y}}|{\varvec{x}}) = \prod ^M _{i=1} \left[ \frac{{\left( \sum ^{N^2} _{j=1} C_{ij} x_j \right) }^{y_i}}{y_i !} \exp \left( -\sum ^{N^2} _{j=1} C_{ij} x_j \right) \right] , \end{aligned}$$

where, $x_j$ is the radioactivity concentration in the *j*-th pixel of matrix size $N^2$, $y_i$ is the projection data in the *i*-th projection data of *M*, and $C_{ij}$ is the detection probability from the *j*-th image pixel to the *i*-th projection data. The ML method estimates ${\varvec{x}}$ maximizing the logarithm of Equation (1), which is the log-likelihood function $L\left( {\varvec{x}}\right)$, as follows [17]:

2 $$\begin{aligned} L\left( {\varvec{x}}\right) = \sum ^M _{i=1} \left[ y_i \ln \left( \sum ^{N^2} _{j=1} C_{ij} x_j \right) - \sum ^{N^2} _{j=1} C_{ij} x_j \right] . \end{aligned}$$

The MAP-EM algorithm, based on Bayes’ theorem, maximizes the log-likelihood function (2) and the prior function. Bayes’ theorem is as follows:

3 $$\begin{aligned} P\left( {\varvec{x}}|{\varvec{y}}\right) = \frac{P\left( {\varvec{y}}|{\varvec{x}}\right) \cdot P\left( {\varvec{x}}\right) }{P\left( {\varvec{y}}\right) }, \end{aligned}$$

where, $P\left( {\varvec{x}}\right)$ is the prior function and is assumed to be a Gibbs distribution [4].

4 $$\begin{aligned} P\left( {\varvec{x}}\right) \propto \exp \left( -\eta U\left( {\varvec{x}}\right) \right) , \end{aligned}$$

where, $U\left( {\varvec{x}}\right)$ is the energy function, and $\eta$ is a regularization parameter. Using Equations (2), (3), and (4), the reconstruction problem of the MAP-EM algorithm is the logarithm of the posterior probability shown in the following equation:

5 $$\begin{aligned} \arg \max _{{\varvec{x}}} \ln P\left( {\varvec{x}}|{\varvec{y}}\right)&= \arg \max _{{\varvec{x}}} \left\{ L\left( {\varvec{x}}\right) + \ln P\left( {\varvec{x}}\right) \right\} \\&= \arg \max _{{\varvec{x}}} \left\{ \sum ^M _{i=1} \left[ y_i \ln \left( \sum ^{N^2} _{j=1} C_{ij} x_j \right) - \sum ^{N^2} _{j=1} C_{ij} x_j \right] - \eta U\left( {\varvec{x}}\right) \right\} .~~ \end{aligned}$$

The MAP-EM algorithm is derived to solve problem (5) and is shown in the following equation [3]:

6 $$\begin{aligned} x ^{k+1} _j = \frac{x ^k _j}{\sum \limits ^M _{i=1} C_{ij} + \eta \frac{\displaystyle \partial }{\displaystyle \partial x_j} U\left( {\varvec{x}}\right) \bigg |_{{\varvec{x}} = {\varvec{x}} ^k}} \sum ^M _{i=1} \frac{y_i C_{ij}}{\sum \limits ^{N^2} _{n=1} C_{in} x ^k _n}, \end{aligned}$$

where, *k* is the number of main iterations. In the TV-EM algorithm, the TV norm is used for $U\left( {\varvec{x}}\right)$, and is expressed as follows [4]:

7 $$\begin{aligned} U_{\text{TV}} \left( {\varvec{x}}\right) = \sum _{s,t} \sqrt{\left( x_{s+1,t} - x_{s,t} \right) ^2 + \left( x_{s,t+1} - x_{s,t} \right) ^2}, \end{aligned}$$

where, *s* and *t* are the pixel numbers in the horizontal and vertical directions of the two-dimensional image, respectively. As the TV norm is not a differentiable function, an approximate formula for the partial derivative of the TV norm by $x_{s,t}$ was used.

8 $$\begin{aligned} \frac{\partial U_{\text{TV}}\left( {\varvec{x}}\right) }{\partial x_{s,t}}&= \frac{x_{s,t} - x_{s-1,t}}{\sqrt{\left( x_{s,t} - x_{s-1,t}\right) ^2 + \left( x_{s-1,t+1} - x_{s-1,t}\right) ^2 + {\varepsilon }^2}} \\&+ \frac{x_{s,t} - x_{s,t-1}}{\sqrt{\left( x_{s+1,t-1} - x_{s,t-1}\right) ^2 + \left( x_{s,t} - x_{s,t-1}\right) ^2 + {\varepsilon }^2}} \\&- \frac{x_{s+1,t} + x_{s,t+1} - 2x_{s,t}}{\sqrt{\left( x_{s+1,t} - x_{s,t}\right) ^2 + \left( x_{s,t+1} - x_{s,t}\right) ^2 + {\varepsilon }^2}}, \end{aligned}$$

where, $\varepsilon$ is an artificial parameter to ensure the differentiability around a point where $U\left( {\varvec{x}}\right) =0$ and usually set to a small value. A previous study mentioned that $\varepsilon$ should be set to 1% or less of the expected maximum value of ${\varvec{x}}$, and that TV-EM is not sensitive to $\varepsilon$ [4]. Therefore, in this study, $\varepsilon$ was set to 0.001.

BSREM is a relaxed type of regularization reconstruction method, which is an extension of RAMLA to the regularized case and is derived by differentiating $L\left( {\varvec{x}}\right)$ and $P\left( {\varvec{x}}\right)$ [9]. The BSREM algorithm is expressed as follows:

9 $$\begin{aligned}&x ^{\left( k,q+1/2\right) } _j = x ^{\left( k,q\right) } _j + \lambda _k x ^{\left( k,q\right) } _j \sum _{i\in S_q} C_{ij} \left( \frac{y_i}{\sum ^{N^2} _{n=1}C_{in}x ^{\left( k,q\right) } _j} - 1\right) , \end{aligned}$$

10 $$\begin{aligned}&x ^{\left( k,q+1\right) } _j = x ^{\left( k,q+1/2\right) } _j + \lambda _k x ^{\left( k,q+1/2\right) } _j \eta \frac{\partial }{\partial x_j} U\left( {\varvec{x}}\right) \Big | _{{\varvec{x}} = {\varvec{x}}^{\left( k,q+1/2\right) }} , \end{aligned}$$

11 $$\begin{aligned}&x ^{\left( k+1,0\right) } _j = x ^{\left( k,Q\right) } _j , \end{aligned}$$

where, $\lambda$ is the relaxation parameter, *q* is the number of sub-iterations, $S_q$ represents all elements in the *q*-th sub-iteration, and *Q* is the total number of sub-iterations. As the name implies, the modified-BSREM is a modified version of the BSREM proposed to converge to global solutions under realistic conditions [10]. In the modified-BSREM, the update formula and relaxation parameters are controlled to not exceed the upper and lower bounds to comply with the constraints, and non-negative value processing is included in the algorithm (Table I and II in [10]). The relaxation parameter of the modified-BSREM is given by:

12 $$\begin{aligned} \lambda _k=\frac{\lambda _0}{\gamma k+1}, \end{aligned}$$

where, $\lambda _0$ is the initial value of the relaxation parameter, and $\gamma$ is the rate for decreasing the relaxation parameter $\lambda$ for each main iteration *k*. The user is required to experimentally set $\lambda _0$, $\gamma$, *Q*, and $\eta$ to use the modified-BSREM appropriately. In the modified-BSREM, since the one of the conditions for convergence to optimal solution is $\sum ^{\infty } _{k=0} \lambda _k =\infty$, a reconstruction is almost independent of $\gamma$ [10]. We verified this in a preliminary experiment, and the influence of $\gamma$ on reconstruction is small when $\gamma$ is small enough. Therefore, in this study, $\gamma$ was set at 0.1 and *Q* included at least three projections in each subset [10].

### Proposed method

The image reconstruction method of RAREM was derived by referring to RAMLA and DRAMA using MAP estimation [1–4, 9]. In RAMLA,

13 $$\begin{aligned} g_q \left( {\varvec{x}}\right) = \sum _{i\in S_q} \left[ y_i \ln \left( \sum _{j=1} ^{N^2} {C_{ij} x_j} \right) - \sum _{j=1} ^{N^2} {C_{ij} x_j} \right] , \end{aligned}$$

14 $$\begin{aligned} x_j ^{\left( k,q+1\right) } = x_j ^{\left( k,q\right) } + \lambda _k x_j ^{\left( k,q\right) } \frac{\partial {g_q \left( {\varvec{x}}\right) }}{\partial {x_j}}, \end{aligned}$$

are derived by incorporating the row-action algorithm into the ML estimation in Equation (2), where, $g_q \left( {\varvec{x}}\right)$ is the log-likelihood function $L\left( {\varvec{x}}\right)$ in the row-action algorithm. Equation (14) is equivalent to Equation (9) in the BSREM algorithm. The goal of RAREM is to maximize Equation (5), which is derived from the logarithms of posterior probability $P\left( {\varvec{x}}|{\varvec{y}}\right)$. Therefore, $g_q\left( {\varvec{x}}\right)$ can be rewritten as

15 $$\begin{aligned} g_q \left( {\varvec{x}}\right) = \sum _{i\in S_q} \left[ y_i \ln \left( \sum _{j=1} ^{N^2} {C_{ij} x_j} \right) - \sum _{j=1} ^{N^2} {C_{ij} x_j} \right] - \eta U\left( {\varvec{x}}\right) \end{aligned}$$

and differentiated with respect to $x_j$ to maximize it, the RAREM algorithm is derived as follows:

16 $$\begin{aligned} & x ^{\left( k,q+1\right) } _j = x ^{\left( k,q\right) } _j + \nonumber \\ & \lambda ^{\left( k,q\right) } x ^{\left( k,q\right) } _j \left\{ \sum _{i\in S_q} C_{ij} \left( \frac{y_i}{\sum ^{N^2} _{n=1}C_{in}x ^{\left( k,q\right) } _j} - 1\right) - \eta _k \frac{\partial }{\partial x_j} U\left( {\varvec{x}}\right) \Big | _{{\varvec{x}} = {\varvec{x}}^{\left( k,q\right) }}\right\} , \end{aligned}$$

17 $$\begin{aligned} & x_j ^{\left( k+1,0\right) } = x_j ^{\left( k,Q\right) }. \end{aligned}$$

**Fig. 1:**
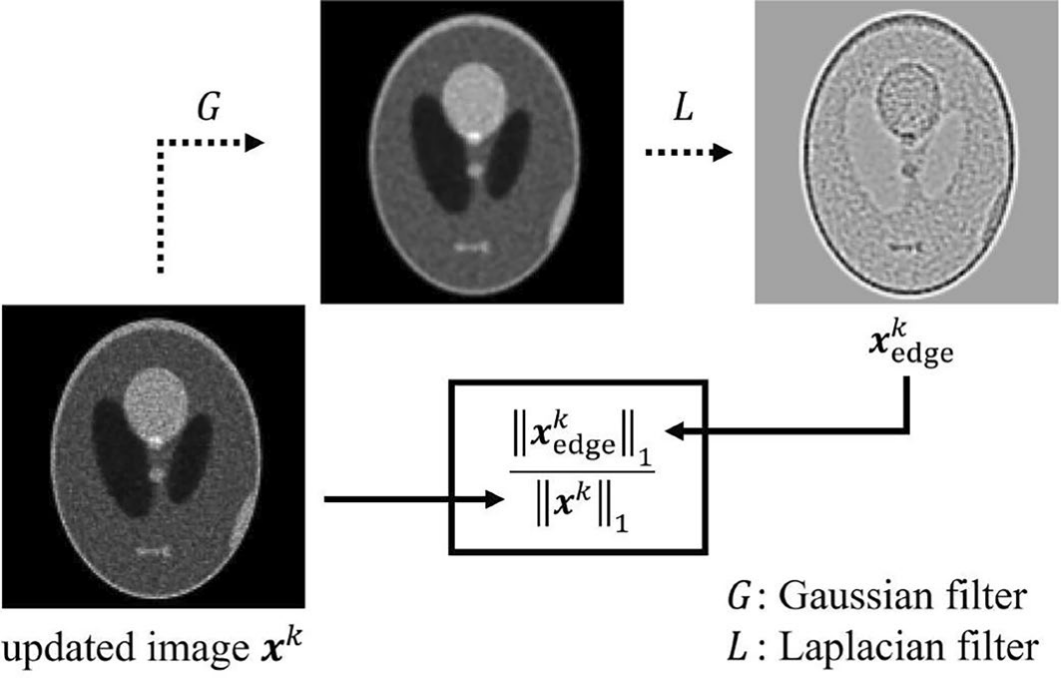
The schematic calculation of $E_k$. $E_k$ is the ratio of the sum of the absolute pixel value of the updated image ${\varvec{x}}^k$ to that of the edge image ${\varvec{x}}^k _{\text{edge}}$

In RAREM, the number of subsets is set to the maximum, as in RAMLA and DRAMA, and each parameter is calculated automatically considering the objective structure and acquisition conditions. In this study, the TV norm in Equation (8) is used for regularization term $\partial U_{\text{TV}} \left( {\varvec{x}}\right) /\partial x_{s,t}$. The regularization parameter $\eta _k$ is calculated by the following equation:

18 $$\begin{aligned} \eta _k = \frac{1}{E_k} \times \left\{ 0.05 \times \left( 1+ A_{\text{proj}}\right) + 0.3 \times A_{\text{count}} \right\} , \end{aligned}$$

19 $$\begin{aligned} A_{\text{proj}} = \max \left\{ \log _{10} { \frac{M_{\mathrm {N_q}}}{M} }, 0 \right\} , \end{aligned}$$

20 $$\begin{aligned} A_{\text{count}} = \max \left\{ \log _{10} \left( \frac{N}{128} \times \frac{10^7}{T}\right) , 0 \right\} , \end{aligned}$$

where, $M_{\mathrm {N_q}}$ is the number of projections that satisfy the Nyquist-Shannon sampling theorem, and *T* is the total number of counts. $\max \left( a,b\right)$ indicates the calculation that selects a higher value *a* or *b*. $A_{\text{proj}}$ can be thought of as the term relating to the number of projections, and $A_{\text{count}}$ as the term relating to the counts. $E_k$ is the evaluation term of the object structure. A schematic of the calculation of $E_k$ is shown in Fig. 1, $E_k$ is calculated using the following equation:

21 $$\begin{aligned} E_k = 100 \times \frac{\big \Vert {\varvec{x}}_{\text{edge}} ^k\big \Vert _1}{\big \Vert {\varvec{x}}^k\big \Vert _1}, \end{aligned}$$

22 $$\begin{aligned} {\varvec{x}} ^k _{\text{edge}} = \left( {\varvec{x}} ^k \otimes G \right) \otimes L, \end{aligned}$$

where, $\big \Vert {\varvec{x}}_{\text{edge}} ^k\big \Vert _1$ is the L1 norm of the updated image processed by the Gaussian filter *G* and Laplacian filter *L* for the calculation of $E_k$ only. In this study, the standard deviation $\sigma$ of the Gaussian filter, with a kernel size of $5 \times 5$, was set to the following equation:

23 $$\begin{aligned} \sigma = 0.4 \times \left\{ 1+\log _{10} \left( \frac{120}{M}\right) \right\} \times \left( \frac{10^4}{T/M}\right) ^{1/2}. \end{aligned}$$

All of the pixel values of the initial image ${\varvec{x}}^0$ were 1.0, but the image reconstructed by DRAMA was used for ${\varvec{x}}^0 _{\text{edge}}$ with $\text {main iteration} = \left\lfloor \frac{M_{\mathrm {N_q}}}{M} \right\rfloor +1$, where $\lfloor \cdot \rfloor$ is a floor function.

The relaxation parameter $\lambda ^{\left( k,q\right) }$ is calculated by the following equation:

24 $$\begin{aligned} \lambda ^{\left( k,q\right) } = \frac{\beta _0}{\beta _0 + q + \gamma k M} \times \frac{1}{1+\log _{10} {\frac{M_{\text{Nq}}}{M}}} \times \frac{1}{1+\eta _k V_{\text{max}}}. \end{aligned}$$

If the acquisition condition was $M_{\mathrm {N_q}}<M$, $M_{\mathrm {N_q}}/M$ is set to 1.0. Equation (24) can be devided into three factors. The initial factor is the width of the decrease in $\lambda$ utilized in DRAMA, with $\gamma$ generally set to 1.0 [2]. $\beta _0$ is the parameter calculated by the average of geometrical coefficients among the projection lines in PET and approximately balances the noise propagation from all subsets at the end of each main iteration [2]. In other imaging modalities, $\beta _0$ is calculated by approximate expression [18, 19].

25 $$\begin{aligned} \beta _0 \approx 0.72 \times \frac{1}{s_{\text{fwhm}}} \frac{N^{1.4}}{M^{0.4}}, \end{aligned}$$

where, $s_{\text{fwhm}}$ is defined as the FWHM of the post-smoothing filter, however, since this study did not use the post-smoothing filter, the TV norm is assumed to be the Gaussian filter with $\sigma = 1.3$. $s_{\text{fwhm}}$ was calculated by the following equation, which relates FWHM to standard deviation $\sigma$:

26 $$\begin{aligned} \text{FWHM} = 2 \sigma \sqrt{2\ln 2}, \end{aligned}$$

resulting in $s_{\text{fwhm}} = 3.06$. The second factor in Equation (24) is the factor taking into account the number of projections. The third factor in Equation (24) is to satisfy the non-negativity constraint in the RAREM algorithm, which contributes to the fact that RAREM does not require non-negative constraints and the process of replacing negative value with non-negative value in effect. To derive this factor, Equation (16) is transformed:

27 $$\begin{aligned} & x^{\left( k,q+1\right) } _j =  \\ & x ^{\left( k,q\right) } _j \Bigg [ 1 + \left. \lambda ^{\left( k,q\right) } \left\{ \sum _{i\in S_q} C_{ij} \left( \frac{y_i}{\sum ^{N^2} _{n=1}C_{in}x ^{\left( k,q\right) } _j} - 1\right) - \eta _k \frac{\partial }{\partial x_j} U\left( {\varvec{x}}\right) \Big | _{{\varvec{x}} = {\varvec{x}}^{\left( k,q\right) }}\right\} \right] . \end{aligned}$$

As $x ^{\left( k,q\right) } _j$ is a non-negative value, we need to consider only the inside of the square brackets in Equation (27). Moreover, since $\lambda ^{\left( k,q\right) }$ is also a non-negative value, we consider when the inside of the curly brackets in Equation (27) is a minimum value. The minimum value for the first term inside the curly brackets is $-1$, because both $C_{ij}$ and $y_i$ are also non-negative values. The minimum value for the second term inside that is when $\frac{\partial }{\partial x_j} U\left( {\varvec{x}}\right) \big | _{{\varvec{x}} = {\varvec{x}}^{\left( k,q\right) }}$ is a maximum value $V_{\text{max}}$. From the above, we can obtain the following inequality to ensure that $x ^{\left( k,q+1\right) } _j$ is a non-negative value:

28 $$\begin{aligned} 1+\lambda ^{\left( k,q\right) } \left( -1-\eta _k V_{\text{max}}\right) > 0. \end{aligned}$$

Finally, inequality (28) is transformed, and the third factor in Equation (16) is obtained using the following inequality:

29 $$\begin{aligned} \lambda ^{\left( k,q\right) } < \frac{1}{1+\eta _k V_{\text{max}}}. \end{aligned}$$

The $V_{\text{max}}$ of the partial derivative of the TV norm (8) is $2+\sqrt{2}$, and the proof of this is shown in Appendix A.

## Experimental methods

### Simulation condition

**Fig. 2:**
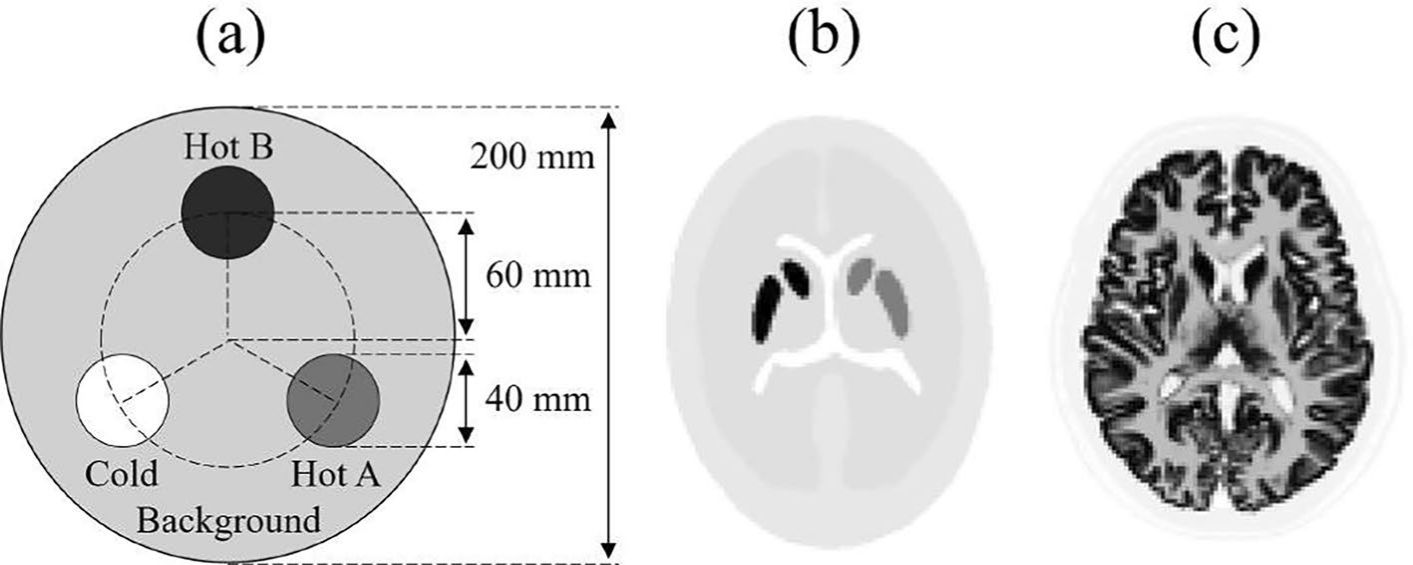
Schematic diagram of phantoms for the simulation experiments. **a** 3D-MAC phantom, **b** IB-20 Advanced (striatum phantom), **c** BrainWeb phantom

In this simulation, a 3-dimensional mathematic phantom (3D-MAC phantom: Nuclear Medicine Section, Japanese Society of Radiological Technology), IB-20 Advanced (Striatum phantom: Kyoto Kagaku Co., Ltd., Kyoto, Japan), and BrainWeb phantom (https://brainweb.bic.mni.mcgill.ca/brainweb/) were used (Fig. 2) [20]. Only the striatum phantom was constructed using the CT data. The components and set radioactivity concentrations of the three phantoms were shown in Table 1. The matrix size and pixel size were $128 \times 128$ and $2.0 ~ {\rm{mm}} \times 2.0 ~ {\rm{mm}}$, respectively. The total projection angle was set to $360^{\circ }$. The total number of projections was varied from 12 to 120. Poisson noise was added to the projection data, varying from $2.5 \times 10^3$ to $5.0 \times 10^4$ counts/projection [21–24].

**Table 1:** The components and the ratio of radioactivity concentration of simulation phantoms

	Radioactivity concentration									
3D-MAC	Background	1	Cold	0	Hot A	2	Hot B	4		
Striatum	Parenchyma	1	Ventricles	0	R striatum	8	L Striatum	4	Other	0.75
BrainWeb	Background	1	WM	5	GM	20				

WM and GM indicate white and gray matter, respectively

### Acquisition conditions for real phantom data

**Fig. 3:**
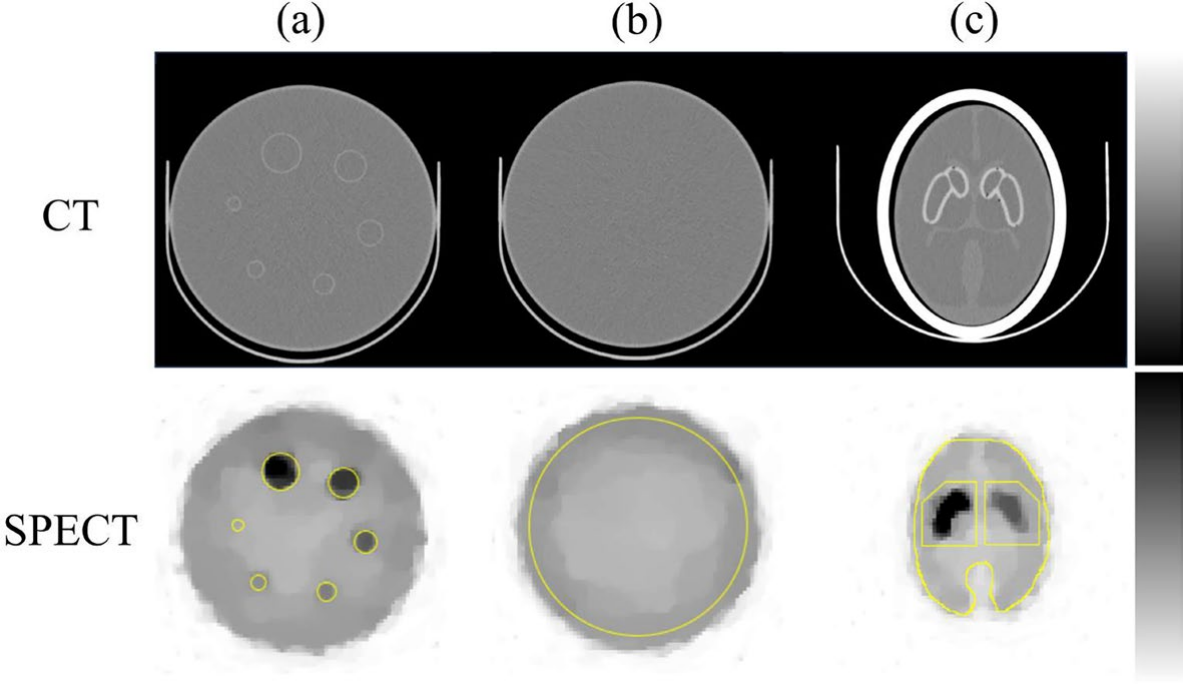
Images of the phantoms used in the real phantom experiment. **a**, **b** show the hot sphere and uniform slices in the Jaszczak phantom, respectively, and **c** show the striatum phantom. The top and bottom rows show the CT and SPECT images, respectively, showing the arrangement of the ROI and VOI. The ROIs at the bottom of **a** were set to the same diameter as those of each of the six hot spheres (9.5, 12.7, 15.9, 19.1, 25.4, and $31.8~{\rm{mm}}$). The ROI at the bottom of **b** was selected as the area containing 85% of the uniform slice. The bottom of **c** denotes the striatum VOI and reference VOI at the striatum level in the striatum phantom.

In this experiment, the Jaszczak phantom (Data Spectrum Corporation, Durham, North Carolina, USA) and IB-20 Advanced (Striatum phantom) were used (Fig. 3). The Jaszczak phantom is a cylindrical phantom containing six hot spheres, with the remaining space being the background region. The diameters of six hot spheres were 9.5, 12.7, 15.9, 19.1, 25.4 and 31.8 mm. It was filled with $^{99\text{m}}$Tc-pertechnetate, which had an approximately 4:1 radioactivity ratio (hot spheres, 26.2 kBq/mL; background, 7.3 kBq/mL). Striatum phantom was also filled with $^{99\text{m}}$Tc-pertechnetate, which had an approximately 8:4:1 radioactivity ratio (right striatum, 56.0 kBq/mL; left striatum, 27.0 kBq/mL; brain parenchyma, 7.0 kBq/mL).

SPECT data were acquired using a Symbia Intevo SPECT/CT system (Siemens Healthcare, Erlangen, Germany) equipped with a low-energy high resolution collimator. All projection data were acquired in step and shoot mode with a circular orbit of $360^{\circ }$, and the radius of rotation was set at $15 ~ {\rm{cm}}$. The number of projections was varied from 30 to 120. The matrix size and pixel size were $128 \times 128$ and $3.3 ~ {\rm{mm}} \times 3.3 ~ {\rm{mm}}$ (scale for enlargement, 1.45), respectively. The energy window was $140 ~ \text{keV} \pm 7.5\%$. The acquisition time was set to be approximately $1.0 \times 10^3 ~ \text{counts}/\left( \text{projection} \times \text{slice}\right)$, a total count of approximately $3.0 \times 10^6$ counts for a $90 ~ {\rm{mm}}$ high striatum phantom [21].

### Image evaluation

Normalized root mean square error (NRMSE) and structural similarity index measure (SSIM) were used to evaluate the image quality of the simulation experiment. The NRMSE, which evaluates the accuracy of the reconstructed images, was used to determine the parameters used in conventional reconstruction methods in the simulation and real experiment in terms of the importance of quantification, and calculated as follows:

30 $$\begin{aligned} \text{NRMSE} \left[ \%\right] = 100 \times \sqrt{\frac{\sum \limits ^{N^2} _{j=1} \left( b_j - a_j\right) ^2}{\sum \limits ^{N^2} _{j=1} a ^2 _j}}, \end{aligned}$$

where, ${\varvec{a}}$ is the reference image, and ${\varvec{b}}$ is the reconstructed image. In the simulation experiment, the original image was used as the reference image. In the real experiment, the projection data obtained by simulation of an imaging nuclear detector (SIMIND), which is one of the Monte-Calro simualtion, under the same geometry as in the real experiment, with low noise and a sufficient number of projections, were reconstructed using ML-EM for the reference image [25, 26]. A smaller NRMSE, which is the difference between the reference and reconstructed images, represents better results.

SSIM is an evaluation index used to evaluate visual assessment close to human subjectivity and is calculated as follows:

31 $$\begin{aligned} \text{SSIM} = \frac{1}{N^2} \sum _{j=1} ^{N^2} \frac{\Big (2 \mu _{ja} \mu _{jb} + c_1\Big ) \Big (2 \sigma _{jab} + c_2 \Big )}{\left( \mu _{ja} ^2 + \mu _{jb} ^2 + c_1\right) \left( \sigma _{ja} ^2 + \sigma _{jb} ^2 + c_2 \right) }, \end{aligned}$$

where, $\mu _{ja}$ and $\mu _{jb}$ are the mean values, $\sigma _{ja}$ and $\sigma _{jb}$ are the standard deviations, and $\sigma _{jab}$ is the covariance in the square window centered on the *j*-th pixel of the images ${\varvec{a}}$ and ${\varvec{b}}$, respectively. The size of the square window is $5\times 5$ [27]. $c_1$ and $c_2$ were calculated as $\left( 0.01L\right) ^2$ and $\left( 0.03L\right) ^2$, respectively, where *L* is the dynamic range of the image. Because the SSIM indicates the similarity between the original and reconstructed images, the closer the reconstructed image is to the original image, the closer the SSIM is to 1.

The contrast recovery coefficient (CRC) and specific binding ratio (SBR) were used to evaluate the experimental results. The CRC is a quantitative index used to evaluate the effect of the reconstruction method on spatial resolution and contrast. The CRC of the Jaszczak phantom is calculated as:

32 $$\begin{aligned} \text{CRC} = \left( \frac{\mu _\text{hot}}{\mu _\text{uni}} - 1\right) \bigg / \left( \frac{\text{True}_\text{hot}}{\text{True}_\text{uni}} - 1\right) , \end{aligned}$$

where, $\mu _\text{hot}$ is the mean value of each hot sphere ROI, and $\mu _\text{uni}$ is that of the uniform region ROI. $\text{True}_\text{hot}$ and $\text{True}_\text{uni}$ are the true activity concentration of each hot sphere ROI and that of the uniform region ROI. The circular ROIs of the hot sphere were set to the same diameter as the sphere diameter, and a uniform circular ROI was selected for the area containing $85\%$ of the uniform slice (Fig. 3). The CRC is the ratio of the true radioactivity contrast to the contrast of the reconstructed image. The closer the contrast ratio of the reconstructed image is to the true contrast ratio, the closer the CRC is to 1.

The SBR ($\mathrm {SBR_{Bolt}}$) of the striatum, a quantitative index for diagnosis of dementia was calculated as follows:

33 $$\begin{aligned} \text{SBR} = \frac{\frac{Ct_\text{str}}{C_\text{ref}} - V_\text{VOI}}{V_\text{str}}, \end{aligned}$$

where, $Ct_\text{str}$ and $C_\text{ref}$ are the total count of the striatal VOI and counts per volume (${\rm{mL}}$) of the reference VOI, respectively (Fig. 3) [28]. $V_\text{VOI}$ is the volume of the striatum VOI, and $V_\text{str}$ is the standard volume of the striatum ($11.2~{\rm{mL}}$). The SBR is defined as the ratio of the radioactivity concentration in the striatum to that in the background region. The true SBR value varies depending on the administered dose. Here, the true SBR values for the right and left striatum were 7.00 and 2.86, respectively. The SBR was used to evaluate the clinical applications of RAREM.

### Statistical analysis

The NRMSE and SSIM were compared between TV-EM and RAREM and modified-BSREM and RAREM, respectively, using the Mann–Whitney U test. Differences were considered statistically significant when *p*-values were less than 0.05.

### Hyperparameter selection of conventional reconstruction method

Considering the importance of quantification in nuclear medicine, the parameters with the best NRMSE values for all acquisition conditions were selected for each conventional reconstruction method. $\lambda _0$ and $\eta$ were varied in nine steps (0.2–1.0) and 10 steps (0.001–0.01, 0.01$-$0.1, or 0.1$-$1.0) in each search, respectively [1]. If the maximum or minimum value of the varied parameter range indicated the best NRMSE, the optimal parameter was determined after three additional steps.

In the real experiment, because the original image could not be obtained, a reference image for the calculation of the NRMSE was created by SIMIND. The projection data of the reference image was obtained under the same geometry as that in the real experiment, with a sufficient number of counts and projections. ML-EM was used to reconstruct the reference images.

## Results

### Simulation experiment

**Fig. 4:**
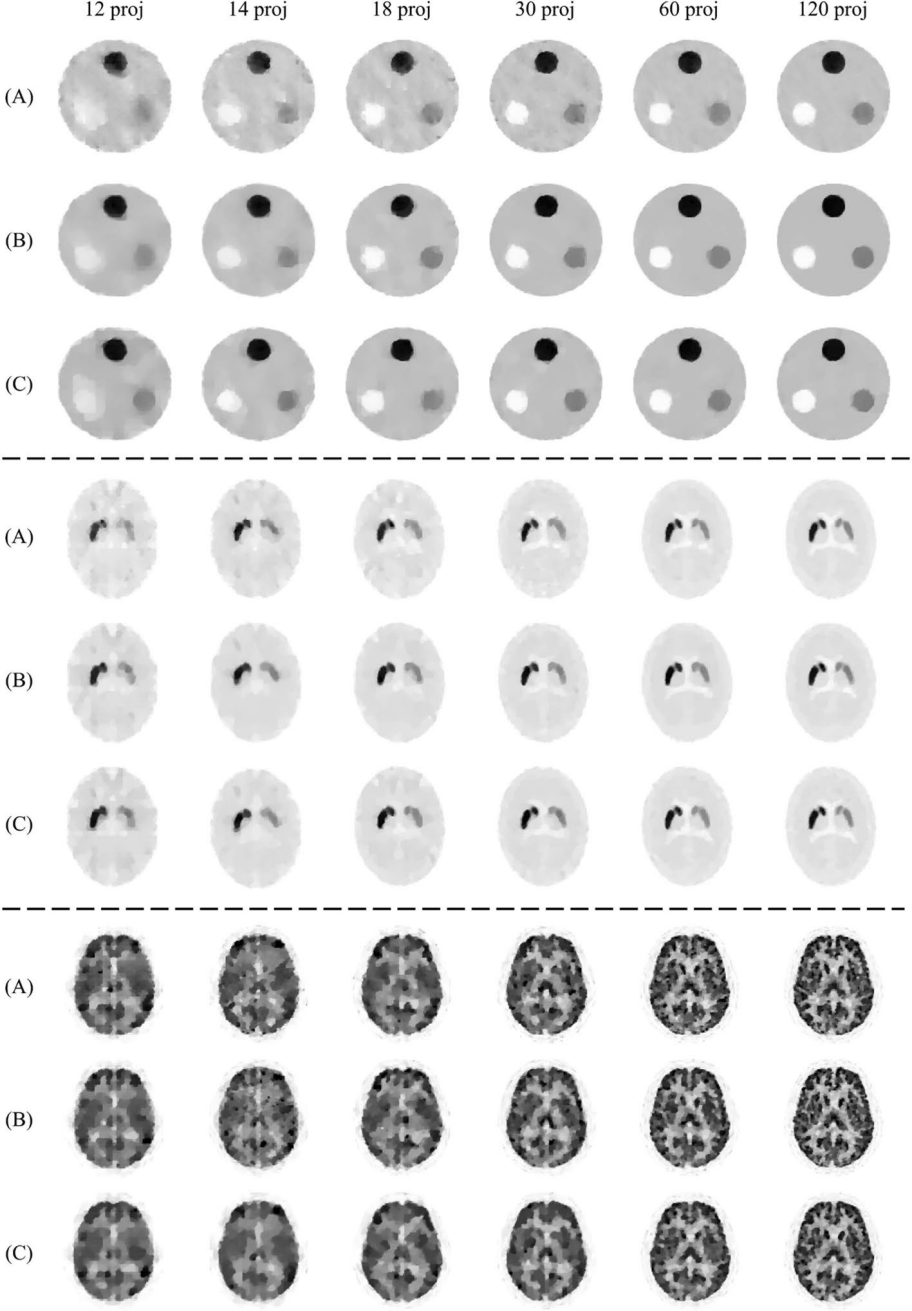
The reconstructed images of the simulation experiment when the counts per projection were $10^4$. **A**, **B**, and **C** are the TV-EM, modified-BSREM, and RAREM, respectively. “proj” indicates the number of projections

The reconstructed images are shown in Fig. 4. The reconstructed images of the 3D-MAC phantom retained the shape of all circular regions under almost all acquisition conditions and reconstruction methods. In contrast, the reconstructed images of the striatum and BrainWeb phantoms did not retain the shape of the structures under poor acquisition conditions, mainly when the number of projections was less than 18. The images of all the phantoms reconstructed by TV-EM were noisier than those reconstructed by the other methods. The reconstructed images showed no significant visual difference between modified-BSREM and RAREM under almost all acquisition conditions.

**Fig. 5:**
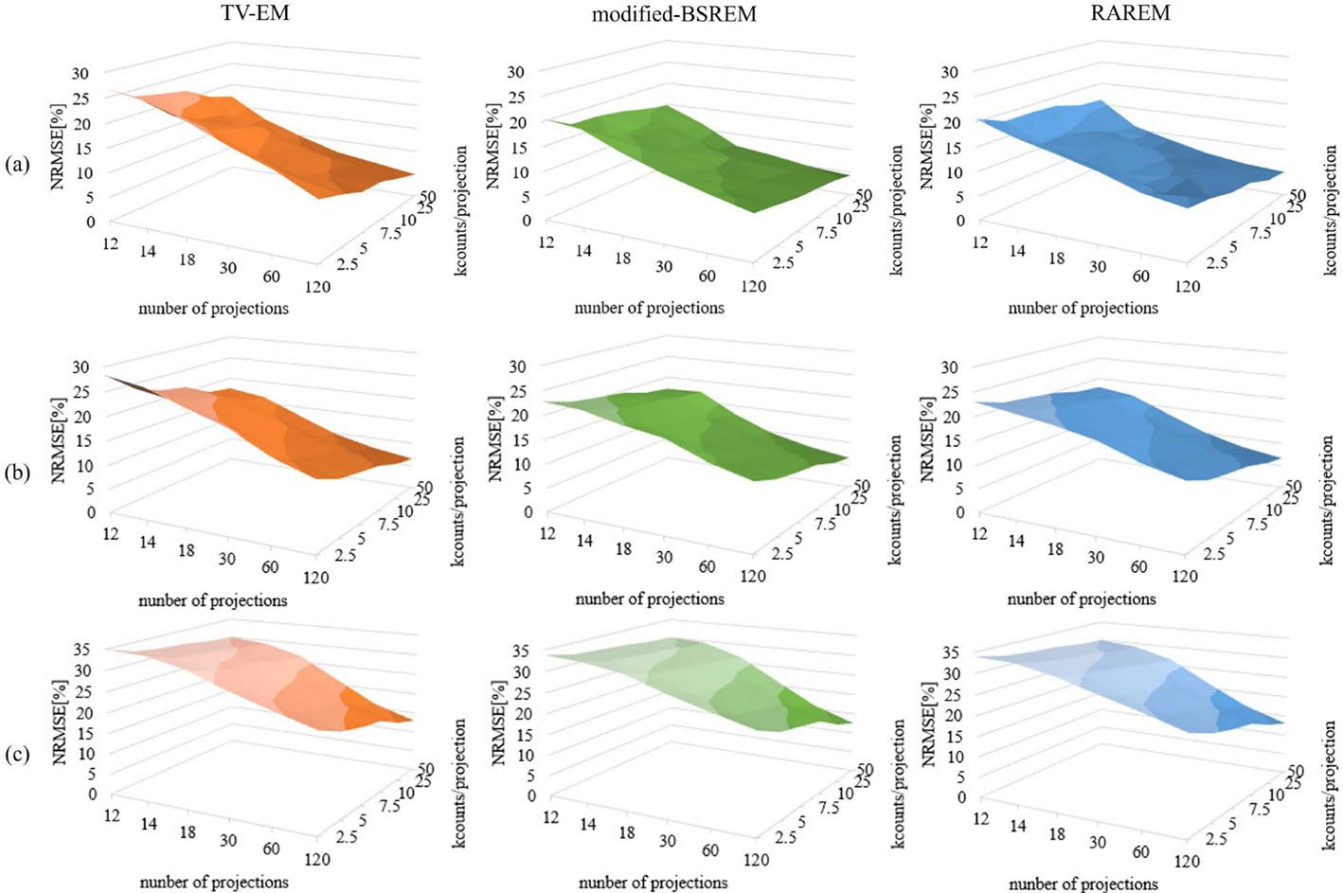
NRMSEs for each reconstruction method and phantom. **a**, **b** and **c** are the 3D-MAC phantom, striatum phantom, and BrainWeb phantom, respectively. “kcounts” indicates $10^3$ counts

**Fig. 6:**
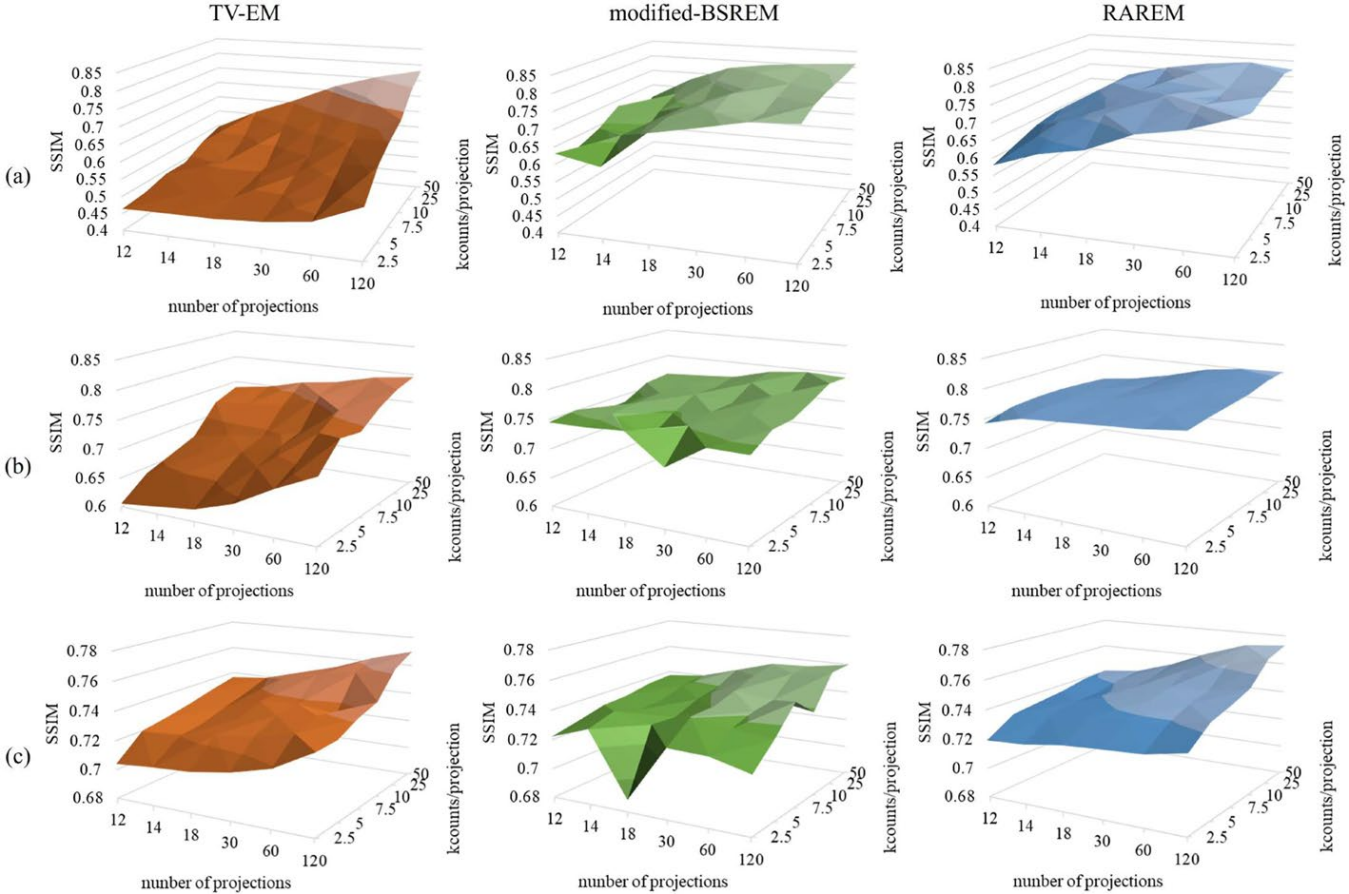
SSIMs for each reconstruction method and phantom. **a**, **b** and **c** are the 3D-MAC phantom, striatum phantom, and BrainWeb phantom, respectively. “kcounts” indicates $10^3$ counts

The NRMSE and SSIM are shown in Figs. 5 and 6, respectively. The NRMSE and SSIM are the mean values of all slices for each acquisition condition. Under almost all acquisition conditions, the NRMSEs of the TV-EM were higher, and the SSIMs were lower than those of the others. This was consistent with the visual assessment presented in Fig. 4. In addition, the NRMSEs were not significantly different between the modified-BSREM and RAREM, whereas the SSIMs of RAREM were slightly higher than those of modified-BSREM, especially when the acquisition conditions were poor. As shown in Fig. 4, modified-BSREM did not outperform RAREM in Figs. 5 and 6. Moreover, the SSIM of the modified-BSREM was low under certain acquisition conditions (such as 18 projection and $2.5 \times 10^3$ counts/projection in the BrainWeb phantom). Contrastingly, those reconstructed using RAREM were stable regardless of the acquisition conditions, even if the number of counts was poor.

**Fig. 7:**
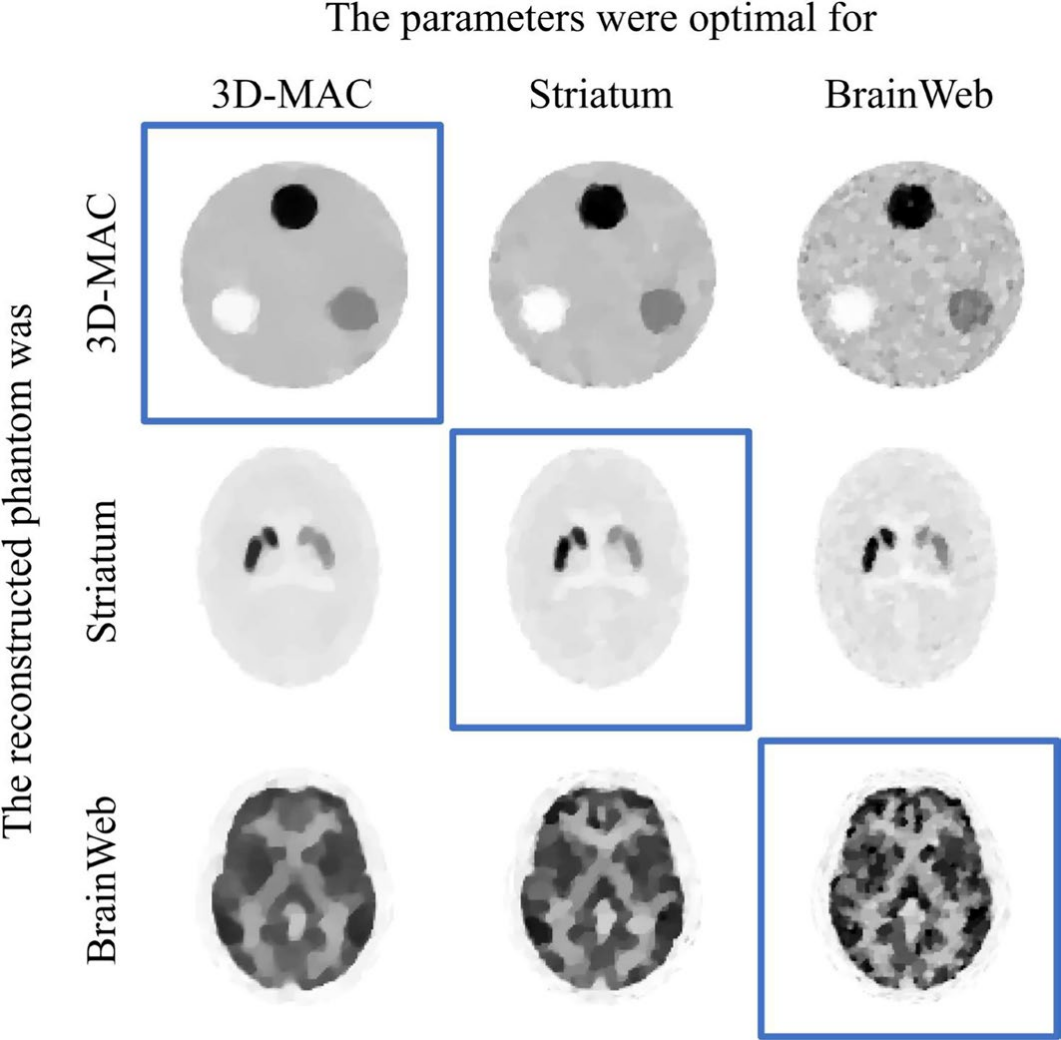
The images reconstructed by modified-BSREM with a different parameter that is optimal for the other phantoms. The acquisition conditions are 60 projections and $5.0 \times 10^3 ~\text{counts} / \text{projection}$. The rows indicate the phantom used, and the columns indicate the reconstruction using the optimal parameters for the noted phantoms. The blue frames indicate the images reconstructed with optimal parameters, and the other images indicate the images reconstructed with non-optimal parameters

The images reconstructed by the modified-BSREM with different parameters that were optimal for the other phantom are shown in Fig. 7. Focusing diagonally from the top left to the bottom right in Fig. 7, the images were reconstructed using the optimal parameters. The reconstructed image of the 3D-MAC phantom using the optimal parameters for the BrainWeb phantom (top right in Fig. 7) was noisier, whereas that of the BrainWeb phantom using the optimal parameters for the 3D-MAC phantom (bottom left in Fig. 7) was excessively smoothed and lost detailed structures. Moreover, in the striatum phantom with optimal parameters for the 3D-MAC phantom (center left in Fig. 7), it was more difficult to visualize the ventricles and showed an overestimation of striatal areas owing to smoothing. Conversely, using the optimal parameters for the BrainWeb phantom (center right in Fig. 7), the image was noisier, and the left striatum structure appeared collapsed.

### Real phantom experiment

**Fig. 8:**
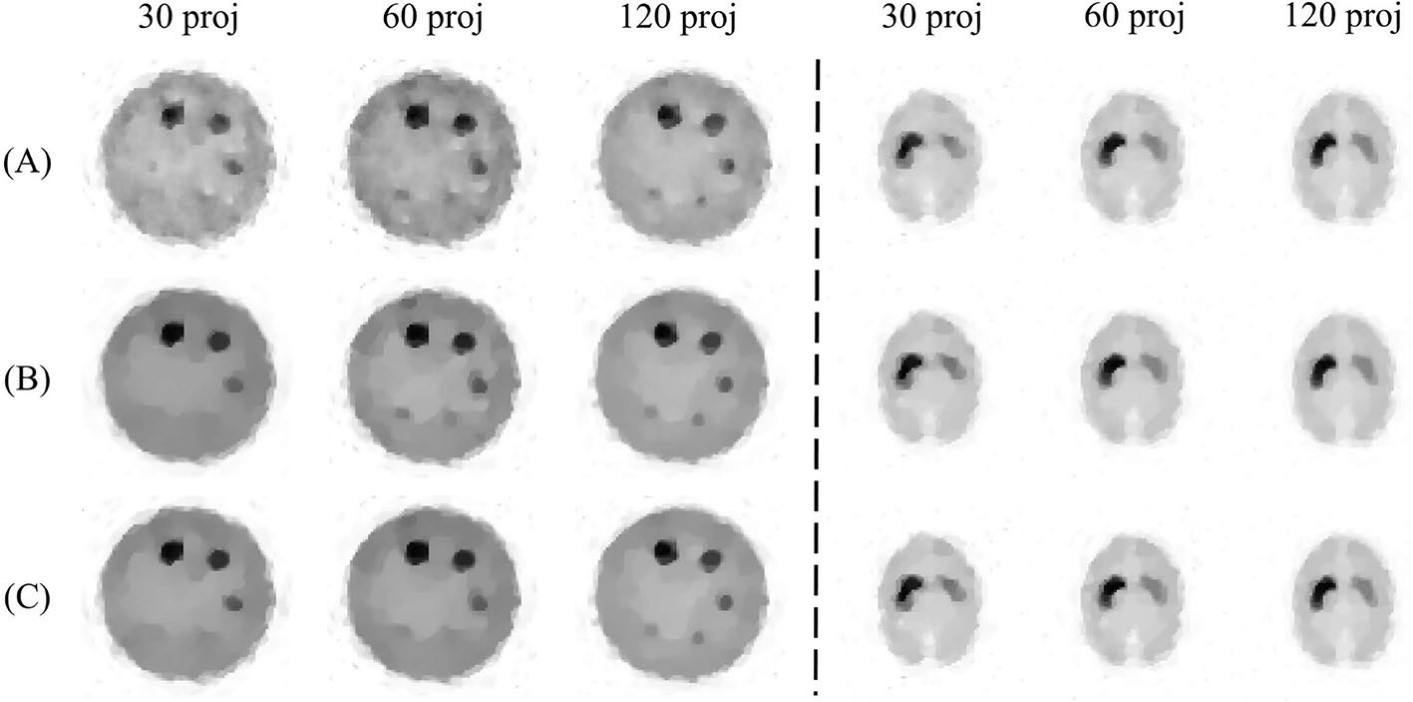
The reconstructed images of the real experiment. The left side is the Jaszczak phantom, and the right side is the striatum phantom. **A**, **B**, and **C** are the TV-EM, modified-BSREM, and RAREM, respectively. “proj” indicates the number of projections

The reconstructed images are shown in Fig. 8. The smallest sphere of the Jaszczak phantom could not be observed under any of the acquisition conditions. The images of the Jaszczak phantom reconstructed by TV-EM exhibited more noise than those reconstructed by the other methods. However, no significant differences existed between modified-BSREM and RAREM in the Jaszczak phantom and between all reconstruction methods in the striatum phantom. The visual results of the real phantom and simulation experiments were the same.

**Fig. 9:**
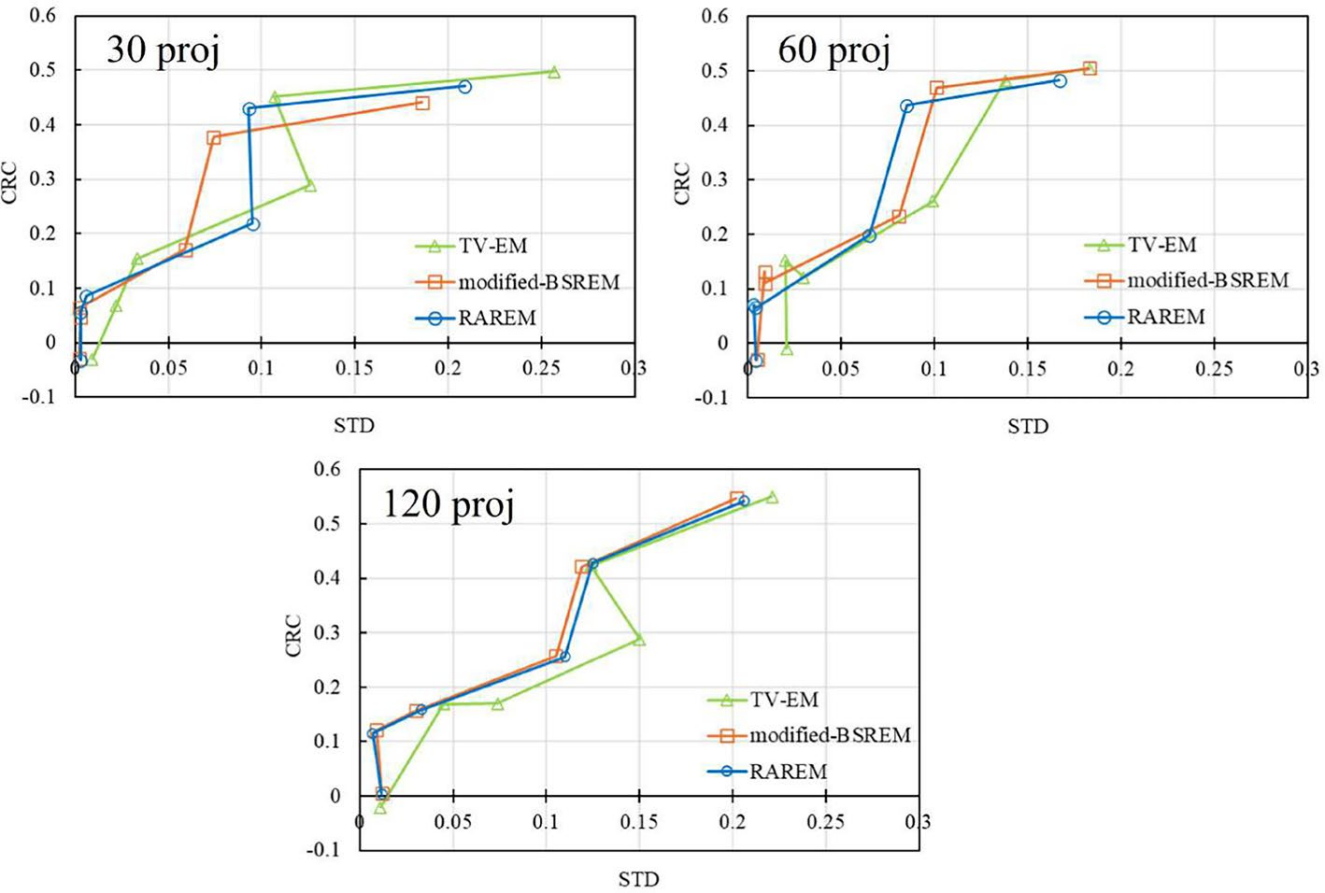
CRCs of the Jaszczack phantom. “proj” and “STD” indicate the number of projections and standard deviation in each ROI, respectively.

**Fig. 10:**
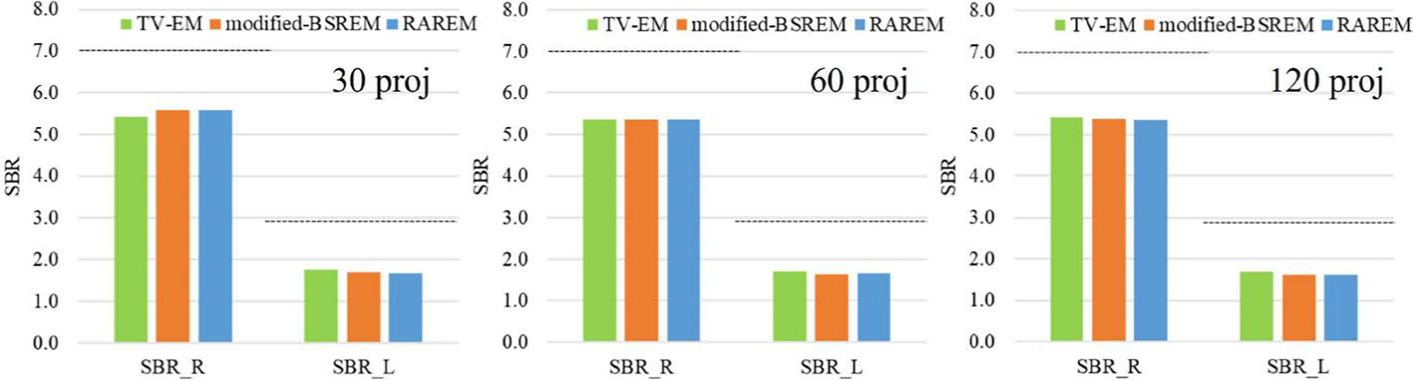
SBRs of the striatum phantom. “proj”, “R”, and “L” indicate the number of projections, the SBR of the right striatum, and the SBR of the left striatum, respectively. The dotted lines indicate the true SBR values

The CRC of the Jaszczak phantom and SBR of the striatum phantom are shown in Figs. 9 and 10, respectively. The CRC was approximately the same for all acquisition conditions and reconstruction methods; however, the standard deviation (STD) in each ROI using TV-EM was slightly higher than that of the other reconstruction methods. The SBR was the same for all the acquisition conditions. In the low-contrast regions (left striatum), the SBRs of all reconstruction methods were approximately $50\%$ of their true values.

## Discussion

RAREM, which we proposed in this study, controlled the regularization parameter $\eta$ and the relaxation parameter $\lambda$ based on the acquisition condition and the complexity of the update image. We evaluated the RAREM algorithm using simulations and real phantom data.

Almost all results using the RAREM were similar to those in the modified-BSREM, where the hyperparameters were optimized. This is attributable to the identical optimization problem (5), and similar solution methods, i.e., gradient method. The statistical analysis results of the SSIM alone for the striatum phantom, where significant differences were found, are shown in Tables 2 and 3. There were no significant differences between conventional methods and RAREM in the other phantoms and evaluations. Under all the acquisition conditions and phantoms, the NRMSE, CRC, and SBR of RAREM were equivalent to those of the conventional methods, and the SSIM of RAREM was better than those of the conventional methods. The regularization parameter was required to set larger value when the acquired data were insufficient, e.g., the number of counts and projections was small [13, 15]. The hyperparameters of RAREM were optimized to achieve good results under all conditions. These results indicate that RAREM can obtain a quantitative performance equivalent to conventional methods and that RAREM can obtain better visual quality images even if the user does not set hyperparameters.

**Fig. 11:**
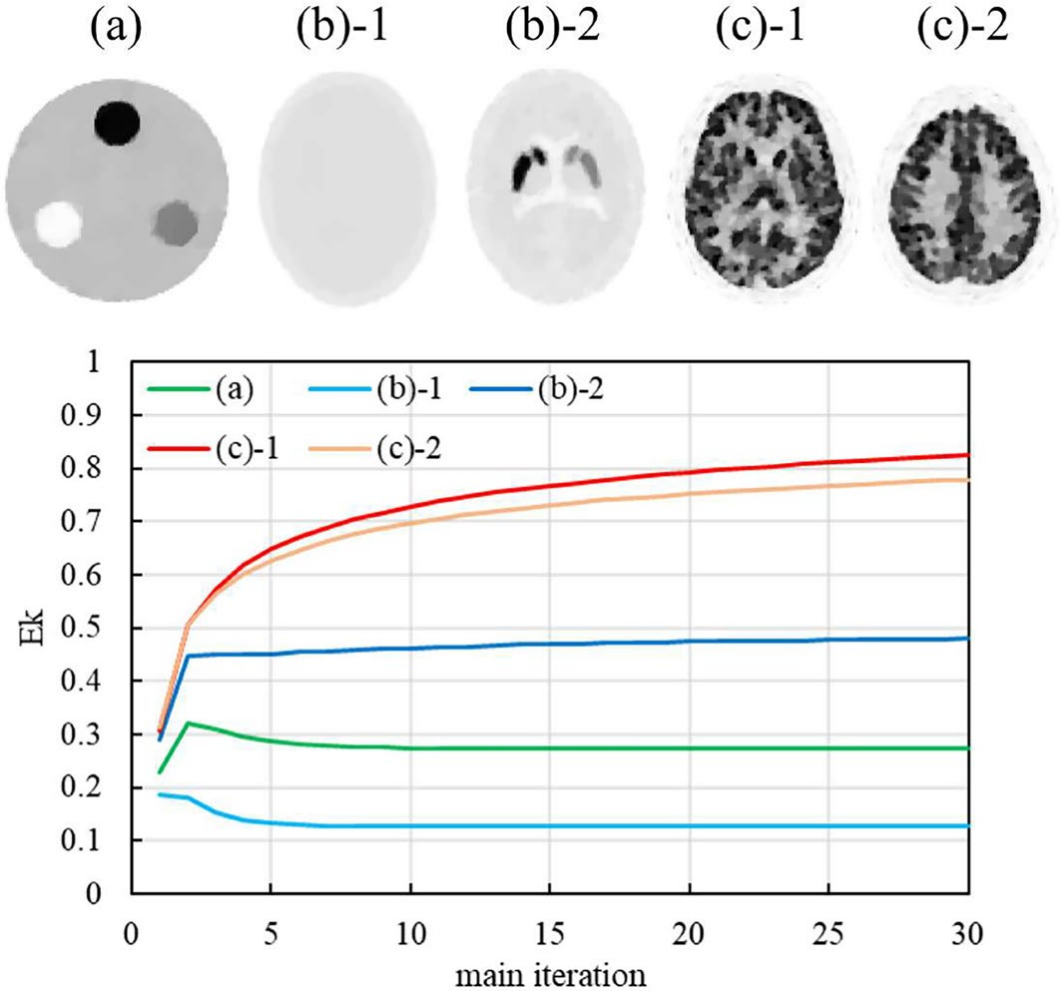
The behavior of $E_k$ reconstructed from 120 projections and $10^4$ counts/projection in RAREM by main iteration

**Fig. 12:**
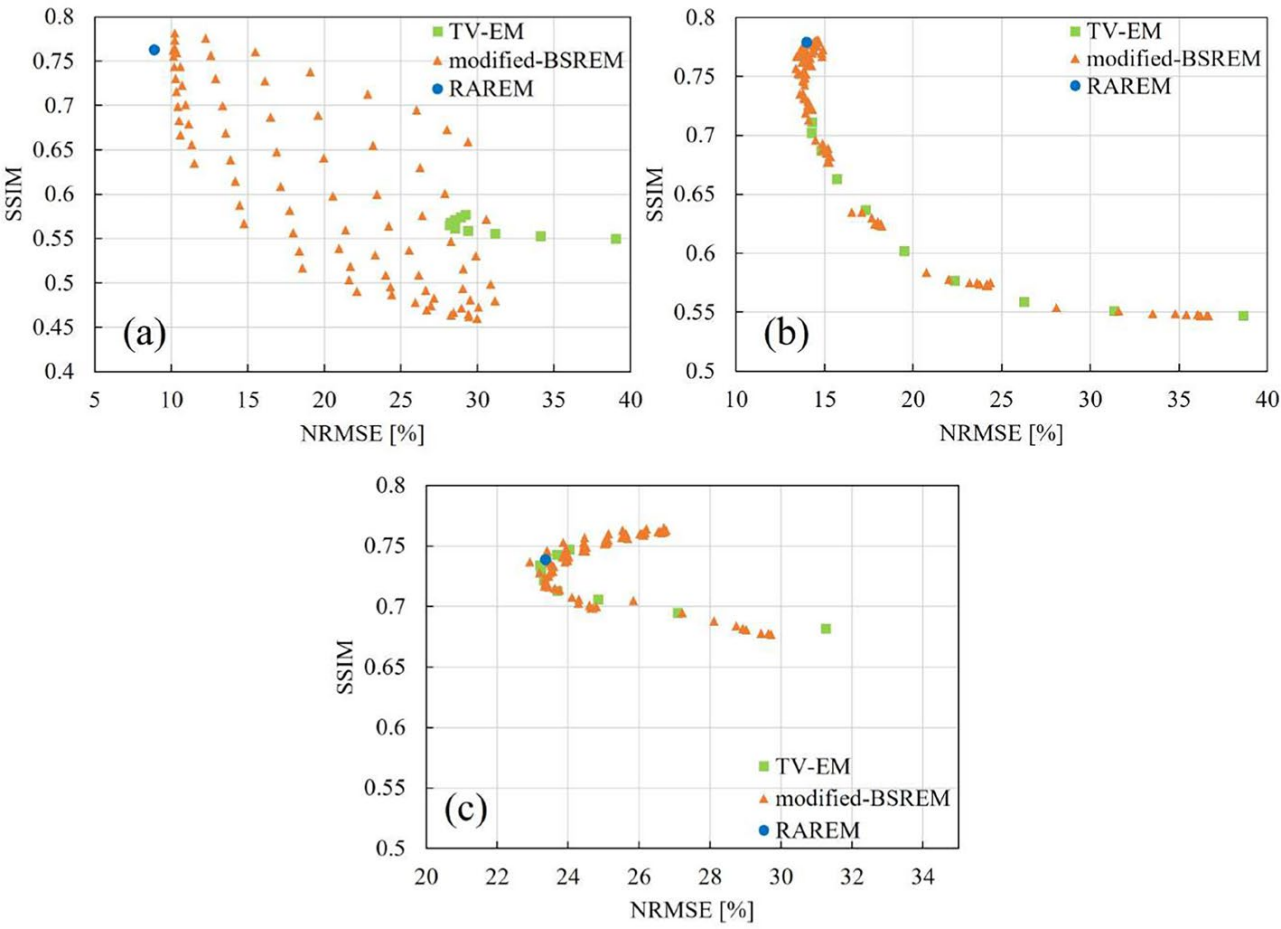
The results of the optimal hyperparameter search under the condition of 60 projections and $5.0 \times 10^3 ~\text{counts} / \text{projection}$ using the TV-EM and modified-BSREM. **a**, **b**, and **c** are the 3D-MAC phantom, Striatum phantom, and BrainWeb phantom, respectively. The range of $\eta$ in TV-EM was from 0.1 to 1.0. The range of $\eta$ in modified-BSREM was from 0.1 to 1.0 in the 3D-MAC phantom and from 0.01 to 0.1 in the other phantoms, and that of $\lambda _0$ was from 0.2 to 1.0

The optimal regularization parameter decreases when the object structure is small or complex because a lower regularization parameter can achieve higher spatial resolution [12, 29]. In addition, the relaxation parameter can control the contribution of noise to update images; therefore, a lower relaxation parameter can suppress noise [1]. However, there are trade-offs between noise suppression and spatial resolution [13]. The values of $E_k$ are shown in Fig. 11. In the simple construction slices ((a) and (b)-1 in Fig. 11), as can be observed in Equations (18) and (24), the $E_k$ had a low value; therefore, $\eta$ and $\lambda$ had high and low values, respectively. In contrast, in the complicated construction slices ((b)-2, (c)-1, and (c)-2 in Fig. 11), the $E_k$ value was high; therefore, $\eta$ and $\lambda$ were low and high, respectively. In the iterative reconstruction method, low-frequency components (mainly contributing to contrast) are recovered with few updates, whereas high-frequency components (mainly contributing to edges and noise) are recovered with many updates [30, 31]. Moreover, decreasing the regularization parameter iteratively is more effective for converging to a global solution than using fixed parameter [32]. Therefore, in RAREM, the increase of $E_k$ and decrease of $\eta$ iteratively contributed to preserving edges in complicated slices and obtaining good image quality. The results indicate that RAREM can automatically optimize the hyperparameters when evaluating the complexity of the object structure. The results of the optimal hyperparameter search using the conventional methods and the results of RAREM are shown in Fig. 12. The NRMSE and SSIM in TV-EM and modified-BSREM had variable values depending on the hyperparameter, and as shown in Fig. 7, using a non-optimal parameters resulted in poor image quality. Optimizing the parameters for regularized reconstruction methods is challenging and has become a field of active research in PET [12–16]. These studies required a lot of time and computation. However, the results of each study are only applicable to its specific target. In nuclear medicine, the invention of a new radio isotope (RI) has led to the creation of new clinical examinations, and the optimal parameters of modified-BSREM have been determined each time with extensive computation and effort because of many hyperparameters. In SPECT, because of the large number of examination types and acquisition conditions, it is expected that searching for the optimal hyperparameters in all examinations is too difficult. However, RAREM can produce good-quality images that are equivalent to the best image obtained from the conventional reconstruction method, with only one calculation. Because RAREM has a similar number of parameters as modified-BSREM, it provides the same flexibility as BSREM while providing images of the same high quality as modified-BSREM without requiring users to experimentally set hyperparameters. RAREM can be applied to any target. Therefore, RAREM is easily applicable in clinical applications.

**Table 2:** Statistical analysis between TV-EM and RAREM for SSIM of the striatum phantom

	kcounts/projection					
projection	2.5	5.0	7.5	10.0	25.0	50.0
12	$$**$$	$$**$$	$$**$$	$$**$$		
14	$$**$$	$$**$$	$$**$$	$$**$$		
18	$$**$$	$$**$$	$$**$$	$$**$$		
30	$$**$$	$$**$$	$$**$$	$$**$$		
60	$$**$$	$$**$$	$$*$$			
120	$$**$$		$$*$$			

“$$*$$” and “$$**$$” indicate $$p<0.05$$ and $$p<0.01$$, respectively

**Table 3:** Statistical analysis between modified-BSREM and RAREM for SSIM of the striatum phantom

	kcounts/projection					
Projection	2.5	5.0	7.5	10.0	25.0	50.0
12						
14						
18		$$**$$				
30	$$**$$					
60						
120						

“$$*$$” and “$$**$$” indicate $$p<0.05$$ and $$p<0.01$$, respectively

## Conclusion

In this study, we proposed an automatically controlled regularized reconstruction method that incorporates acquisition conditions and object structure into the hyperparameters. We evaluated the image quality in the simulation and real phantom experiments, demonstrating that the proposed method performs equivalently or better than conventional regularized methods, with optimized hyperparameters. The proposed method does not require the user to set hyperparameters experimentally and can avoid the investigation of optimal hyperparameters; it is an alternative to conventional regularized methods in clinical.

## Data Availability

The datasets generated during and/or analyzed during the current study are available from the corresponding author on reasonable request.

## Appendix A

In this Appendix, we prove how $V_{\text{max}}$ of the partial derivative of the TV norm (8) is derived. First, $\varepsilon$ is ignored to simplify the equation, because it reduces $V_{\text{max}}$. Next, the first term in Equation (8) is considered. Since $\frac{x_{s,t} - x_{s-1,t}}{\sqrt{\left( x_{s,t} - x_{s-1,t}\right) ^2}} = 1$ and $\left( x_{s-1,t+1} - x_{s-1,t}\right) ^2 \ge 0$, the maximum value of the first term in Equation (8) is 1 when $x_{s-1,t+1} = x_{s-1,t}$. The maximum value of the second term is 1, considering it in the same manner as in the first term. To consider the maximum value of the third term, it is transformed.

34 $$\begin{aligned} \frac{x_{s+1,t} + x_{s,t+1} - 2x_{s,t}}{\sqrt{\left( x_{s+1,t} - x_{s,t}\right) ^2 + \left( x_{s,t+1} - x_{s,t}\right) ^2}} = \frac{\left( x_{s+1,t} - x_{s,t}\right) + \left( x_{s,t+1} - x_{s,t}\right) }{\sqrt{\left( x_{s+1,t} - x_{s,t}\right) ^2 + \left( x_{s,t+1} - x_{s,t}\right) ^2}} \end{aligned}$$

Because the sign of the third term in Equation (8) is negative, it is necessary to consider the minimum value in Equation (34) to consider the maximum value in Equation (8). When $x_{s+1,t}$ and $x_{s,t+1}$ are 0, and $x_{s,t} > 0$, the third term indicates the minimum value because each component of ${\varvec{x}}$ is a non-negative value. Therefore, the minimum value of third term is $-\sqrt{2}$. From the maximum and minimum values of the three terms shown above, the maximum value $V_{\text{max}}$ of equation (8) is $2+\sqrt{2}$.

## Abbreviations

SPECT: Single photon emission computed tomography
CT: Computed tomography
MRI: Magnetic resonance imaging
PET: Positron emission tomography
DRAMA: Dynamic row-action type maximization likelihood algorithm
ML-EM: Maximum likelihood-expectation maximization
OS-EM: Ordered subset-expectation maximization
MAP-EM: Maximum a posteriori-expectation maximization
TV-EM: Total variation-expectation maximization
BSREM: Block sequential regularized expectation maximization
RAREM: Row-action type automatic regularized expectation maximization
FWHM: Full width at half maximum
NRMSE: Normalized root mean square error
SSIM: Structural similarity index measure
SIMIND: Simulation of an imaging nuclear detector
CRC: Contrast recovery coefficient
SBR: Specific binding ratio
ROI: Region of interest
VOI: Volume of interest
